# Critical parameters in cultivation of experimental biofilms using the example of *Pseudomonas fluorescens*

**DOI:** 10.1007/s10856-021-06568-w

**Published:** 2021-08-18

**Authors:** Kirsten Reddersen, André Güllmar, Silke Tonndorf-Martini, Bernd W. Sigusch, Andrea Ewald, Thomas J. Dauben, Karin Martin, Cornelia Wiegand

**Affiliations:** 1 Klinik für Hautkrankheiten, Universitätsklinikum Jena, Jena, Germany; 2 Poliklinik für Konservierende Zahnheilkunde und Parodontologie, Universitätsklinikum Jena, Jena, Germany; 3 Lehrstuhl für Funktionswerkstoffe der Medizin und Zahnheilkunde, Universitätsklinikum Würzburg, Würzburg, Germany; 4 Lehrstuhl für Materialwissenschaft, Otto-Schott-Institut für Materialforschung, Jena, Germany; 5 Hans-Knöll-Institut, Leibnitz-Institut für Naturstoff-Forschung und Infektionsbiologie, Jena, Germany

## Abstract

Formation and treatment of biofilms present a great challenge for health care and industry. About 80% of human infections are associated with biofilms including biomaterial centered infections, like infections of prosthetic heart valves, central venous catheters, or urinary catheters. Additionally, biofilms can cause food and drinking water contamination. Biofilm research focusses on application of experimental biofilm models to study initial adherence processes, to optimize physico-chemical properties of medical materials for reducing interactions between materials and bacteria, and to investigate biofilm treatment under controlled conditions. Exploring new antimicrobial strategies plays a key role in a variety of scientific disciplines, like medical material research, anti-infectious research, plant engineering, or wastewater treatment. Although a variety of biofilm models exist, there is a lack of standardization for experimental protocols, and designing experimental setups remains a challenge. In this study, a number of experimental parameters critical for material research have been tested that influence formation and stability of an experimental biofilm using the non-pathogenic model strain of *Pseudomonas fluorescens*. These parameters include experimental time frame, nutrient supply, inoculum concentration, static and dynamic cultivation conditions, material properties, and sample treatment during staining for visualization of the biofilm. It was shown, that all tested parameters critically influence the experimental biofilm formation process. The results obtained in this study shall support material researchers in designing experimental biofilm setups.

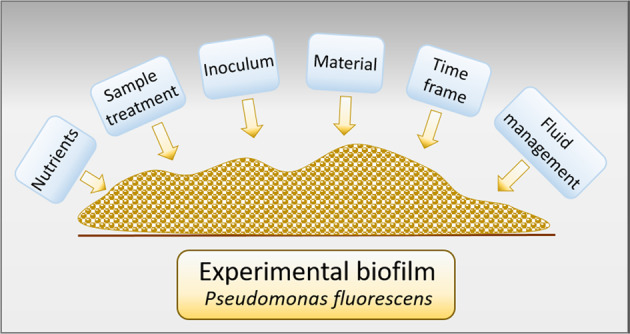

## Introduction

A biofilm is the most common form of naturally occurring microbes [1]. An estimated proportion of 99% of bacteria in natural habitats are sessile and attached as biofilms to a surface [2]. In biofilms, microorganisms are surrounded by an extracellular polymeric matrix (EPM), which accounts for about 85% of the biofilm volume [3]. The EPM creates a protective environment for the microorganisms that enhances the tolerance to harmful external conditions like antibiotics or desiccation by a factor of 10–100 compared to planktonic microorganisms [2]. This sheltering cover is considered the reason that antibiotic treatment of biofilm infections fails.

Biofilm formation plays an important role in health care and industrial settings. On the one hand, beneficial biofilms are used in the industrial production of chemicals like ethanol and vinegar. On the other hand, there are harmful effects of biofilms in production processes that cause food and drinking water contamination, metal surface corrosion, biofouling, and clogging [4]. In human health care, over 80% of all microbial infections in the body are biofilm associated [5]. This includes indwelling medical device infections of e.g. prosthetic heart valves, central venous catheters, or urinary catheters, and diseases like periodontitis, dental caries, cystic fibrosis pneumoniae, chronic urinary tract infections, infections on contact lenses, and chronic wound infections [2, 3, 6, 7]. Biomaterial implants are considered as ´microbial time bombs´, since biomaterial centered infections can happen anytime and treatment is very difficult [8]. A promising approach to prevent biofilm formation on biomaterials is optimizing the physico-chemical properties of biomaterial surfaces. Intelligent material design can reduce attractive forces between biomaterial and bacteria and hence, minimize initial microbial growth.

Research on biofilm development, treatment, or eradication plays a key role in antimicrobial strategies involving a variety of scientific disciplines, like medical material research, anti-infectious research, plant engineering, or wastewater treatment. Biofilm models are important tools to increase the knowledge of the underlying mechanisms of biofilm formation and to study the complex systems under controlled conditions [9]. Although a variety of in vitro and in vivo models exist [10], there is a lack of standardization of experimental protocols [11]. There is no ideal laboratory biofilm model that is representative for all biofilms and suitable for all research questions [2]. Further, the complexity of the subject makes it difficult to decide upon the appropriate biofilm model for the particular task.

In this study, a number of experimental conditions critical for material research were investigated that influence biofilm formation and biofilm stability. This includes experimental time frame, nutrient supply, inoculum concentration, static and dynamic fluid management conditions, material properties, and sample treatment for visualization. The aim of this study is to point out important factors for cultivation of experimental biofilms using the non-pathogenic model strain of *Pseudomonas fluorescens* and to identify critical parameters for designing experimental setups in material research.

## Materials and methods

In this study, different critical parameters that influence the adherence and maturation of experimental biofilm were examined. The parameters were selected with regard to their relevance for testing bacteria/material interactions. The following critical parameters were investigated:

- Time frame of bacterial adherence
- Nutrient supply
- Inoculum concentration
- Fluid management during incubation
- Material properties
- Sample treatment for visualization

### Time frame of bacterial adherence

Shaking overnight cultures of *P. fluorescens* (DSM 50090) were grown in Luria-Bertani-medium (LBM, Carl Roth GmbH, Karlsruhe, Germany) at 37 °C to mid-log phase (OD_600_ ≈ 0.5). The culture was adjusted to an OD_600_ of 0.1 in diluted LBM (1:100 in 0.9% NaCl, Fresenius Kabi AG, Bad Homburg, Germany). Sterilized borosilicate coverslips (15 mm diameter, Karl Hecht KG, Sondheim, Germany) were placed in 12-well plates (Greiner Bio One, Frickenhausen, Germany) and incubated with 2 mL inoculum. Samples were incubated for 15, 30, 60, 90, and 120 min at 25 °C in aerobic conditions. After incubation time, samples were rinsed twice with 2 mL phosphate buffered saline (PBS, PeloBiotech, Planegg, Germany) to remove non-adhering microorganisms, transferred to a fresh 12-well plate, and stained for 15 min with 1.5 mL SYTO9 (0.00334 mM, Invitrogen, Carlsbad, US)/propidium iodide (PI, 0.0107 mg/mL, Invitrogen, Carlsbad, US) at room temperature (RT) in the dark. After rinsing with 1 mL PBS, samples were incubated for 30 min at RT in the dark with 1.5 mL calcofluor white (0.092 mg/mL, CFW, Sigma-Aldrich, Darmstadt, Germany). After rinsing with 1 mL PBS, samples were covered with mounting oil (Invitrogen, Carlsbad, US) and examined using the fluorescence microscope Axio Scope. A1 (Carl Zeiss AG, Jena, Germany) with appropriate filter blocs. Images were taken with the AxioCam MRc camera (Carl Zeiss AG, Jena, Germany) and analysed using AxioVision 4.9 software (Carl Zeiss AG, Jena, Germany).

Adherence studies up to 72 h were performed in undiluted LBM with inoculum concentration of 2 × 10^6^ CFU/mL. Samples were taken after 2, 4, 6, 8, 10, 12, 16, 24, 48, and 72 h. Medium was changed daily. Quantification with Water-soluble-tetrazolium-1 (WST-1, Roche Diagnostics, Mannheim, Germany) was carried out according to the manufacturer´s instructions. Shortly, the samples were placed into fresh wells and incubated with WST-1 (10% in PBS, Sigma-Aldrich, Darmstadt, Germany) for 30 min. The absorbance was measured at 450 nm using a Tecan spark 20 M plate reader (Tecan Group Ltd., Männedorf, Switzerland).

### Nutrient supply

To study the influence of nutrient supply on biofilm adherence, LBM was applied undiluted, 1:10, 1:100, and 1:1000 diluted in NaCl (0.9%). *P. fluorescens* was incubated with an inoculum density of 7.6 × 10^5^ CFU/mL in 4 cm diameter petri dishes with glass bottom in static conditions for 2, 4, 6, 24, 48, and 72 h. Medium was changed daily. For visualization, samples were rinsed carefully twice with 2 mL NaCl (0.9%), stained with PI (8 µM; Carl Roth GmbH, Karlsruhe, Germany) in paraformaldehyde (PFA, 4%, Carl Roth GmbH, Karlsruhe, Germany) over night at 4 °C in the dark. After incubation, samples were washed twice with 2 mL NaCl (0.9%) and overlaid with NaCl (0.9%). Samples were examined with a confocal laser scanning microscope LSM 510 Meta (Carl Zeiss AG, Jena, Germany) equipped with an EC Plan-Neofluar 20x/0.50 M27 objective (Carl Zeiss AG, Jena, Germany). Propidium iodide labeled bacteria were excited with a DPSS laser (561 nm), and their emission passed a 574-nm longpass filter.

### Inoculum concentration

*P. fluorescens* was incubated on 15 mm diameter borosilicate glass coverslips (Karl Hecht KG, Sondheim, Germany) under static conditions. Inoculum concentration was 2 × 10^3^, 2 × 10^4^, 2 × 10^5^, and 2 × 10^6^ CFU/mL in LBM. Samples were taken after 1, 2, 4, 24, 48, and 72 h. Bacteria were quantified by determination of colony forming units on Columbia-agar plates + 5% sheep blood (bioMérieux, Nürtingen, Germany) after separation and serial dilution.

### Fluid management

To compare static and dynamic growth conditions, shaking overnight cultures of *P. fluorescens* in LBM were adjusted to OD 0.5 and diluted 1:100 in LBM. 1 mL inoculum was incubated in 24 well microtiter plates (MTP, Greiner Bio One, Frickenhausen, Germany) in static and dynamic conditions using a wobble shaker (Titramax 100; Heidolph Instruments GmbH & CO. KG, Schwabach, Germany) at 200 rpm. Samples were taken at 2, 4, 6, 24, 48, and 72 h. Medium was changed after 24 and 48 h. Bacteria were quantified by determination of colony forming units after separation and serial dilution.

The influence of sample rinsing on biofilm growth was examined by incubating 15 mm diameter borosilicate coverslips (Karl Hecht KG, Sondheim, Germany) with inoculum concentrations of 2 × 10^7^ CFU/mL for 24, 48, and 72 h. The biofilm was rinsed once with 2 mL LBM before quantification and during medium change after 24 and 48 h. Quantification was done with WST-1 (10%) as described before.

### Material properties

Borofloat glass coverslips (Borofloat^®^ B33, Jena 4H Engineering GmbH, Jena, Germany), Nunc^TM^ Thermanox^TM^ polyester (Fisher Scientific GmbH, Schwerte, Germany), and poly-L-lysin coated coverslips (Corning^®^ BioCoat™ Poly-L-Lysine, Corning Life Sciences B.V., Amsterdam, The Netherlands) were incubated in 24 well MTP (Greiner bio One, Frickenhausen, Germany) with 1 mL inoculum of 1 × 10^6^ CFU/mL for 24, 48, and 72 h. Samples were stained with 1.5 mL crystal violet solution (1%, Sigma-Aldrich, Darmstadt, Germany) for 10 min. After three washing steps with 1 mL sterile distilled water the remaining dye was solved by SDS (1%, Sigma-Aldrich, Darmstadt, Germany) and absorbance was measured at 590 nm using a Spekol 1200 reader (Analytik Jena, Jena, Germany).

### Sample treatment for visualization

Drying effects during sample treatment were visualized with 24 h biofilm on borosilicate glass coverslips (Karl Hecht KG, Sondheim, Germany) in 12-well MTPs. Samples were incubated with an inoculum of OD_600_ 0.1 in LBM diluted 1:100 in NaCl (0.9%). Staining was done with SYTO9/PI/CFW as described in chapter 2.1. To examine the influence of possible drying effects for visualization of the biofilm during sample treatment, on the one hand samples were kept consequently with a liquid film during staining procedure by treating each well separately, on the other hand removal of liquid during staining was done for the whole plate, enabling individual samples to fall dry for a short time.

### Statistics

Experiments were performed in duplicate and each sample was measured in three replicates.

All values presented are expressed as means ± SD. One-way analysis of variance was carried out to determine statistical significances (Microsoft^®^ Excel 2016). Significant (*p < 0.05), very significant (**p < 0.01), and high significant (***p < 0.001) deviations are indicated.

## Results

### Time frame of bacterial adherence

**Fig. 1:**
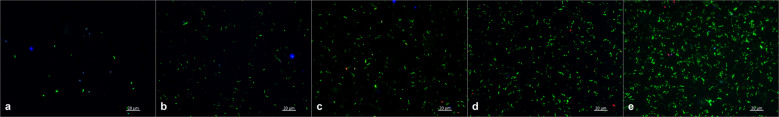
Adherence of *P. fluorescens* to the borosilicate glass substrate was determined after **a** 15 min, **b** 30 min, **c** 60 min, **d** 90 min, and **e** 120 min incubation in LBM 1:100 diluted in 0.9% NaCl. Samples were rinsed after incubation and between staining steps to select only adhering microorganism. Staining of the biofilm was done with SYTO9/PI/CFW

During incubation of *P. fluorescens* (*P. fluorescens*) on a glass substrate in 1:100 diluted Luria-Bertani medium (LBM), adherence of the microorganisms was already visible after 15 min (Fig. 1a). The adhering density amplified steadily in the first 120 min (Fig. 1, b–d) resulting in evenly distributed adhering living bacteria on the surface after 2 h incubation (Fig. 1e).

**Fig. 2:**
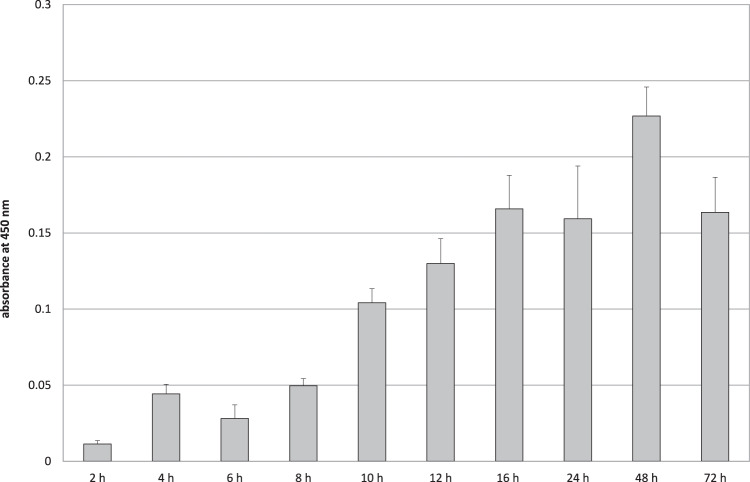
Biomass of *P. fluorescens* biofilm after up to 72 h incubation was quantified by cell proliferation reagent WST-1. Data are given as mean ± standard deviation

Further incubation of *P. fluorescens* for 72 h resulted in an increase of biofilm biomass during the first 16 h (Fig. 2). After that incubation time, an equilibrium between growing biomass and detachment of the biomass during medium change was achieved. Additionally, with growing biofilm the metabolism of the basal cells decreases, which may explain decreasing absorbance values after 72 h incubation. The maximum amount of biomass in this study was detected after 48 h.

### Nutrient supply

**Fig. 3:**
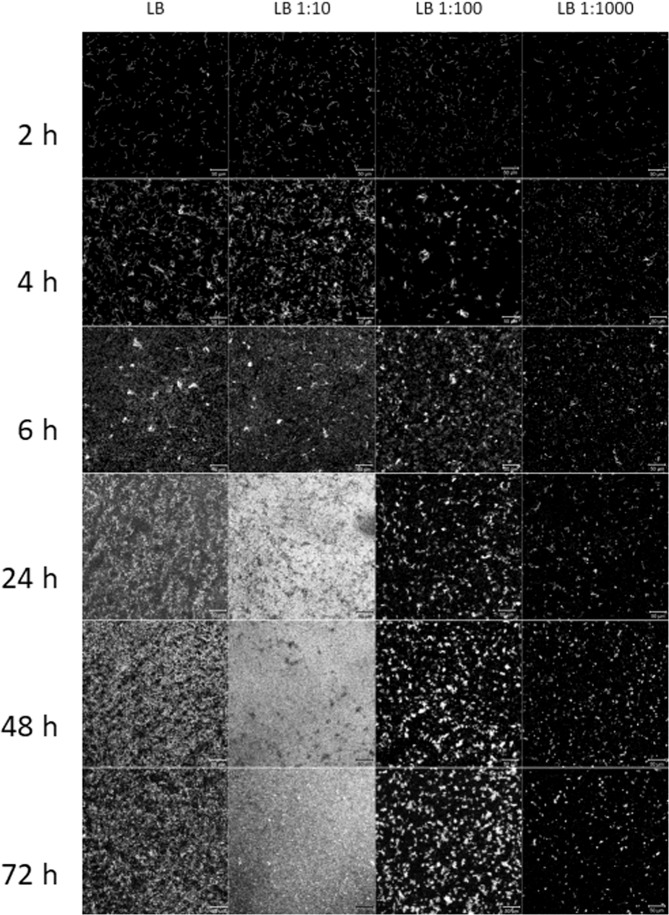
Influence of nutrient supply on biofilm formation of *P. fluorescens* during 72 h incubation. LBM was applied undiluted, 1:10, 1:100, and 1:1000 diluted in 0.9% NaCl. Staining of the biofilm was done with PFA/PI

The influence of nutrient supply on biofilm formation during 72 h was studied by diluting LBM up to 1:1000 in 0.9% NaCl (Fig. 3). After 4 h incubation, the difference in nutrient supply could already be seen with the highest density of adhering microorganisms in undiluted medium and a decreasing density of microorganisms with rising dilution of the medium. In undiluted LBM with high nutrient supply, a strong bacterial growth was observed with biofilm floats and excessive formation of mucus. Incubation with medium nutrient supply in 1:10 diluted LBM lead to slow growth and formation of a stable biofilm on the material surface without excessive mucus formation. Further nutrient reduction in 1:100 and 1:1000 diluted LBM resulted in very slow bacterial growth with no macroscopically visible biofilm formation after 72 h.

### Inoculum concentration

**Fig. 4:**
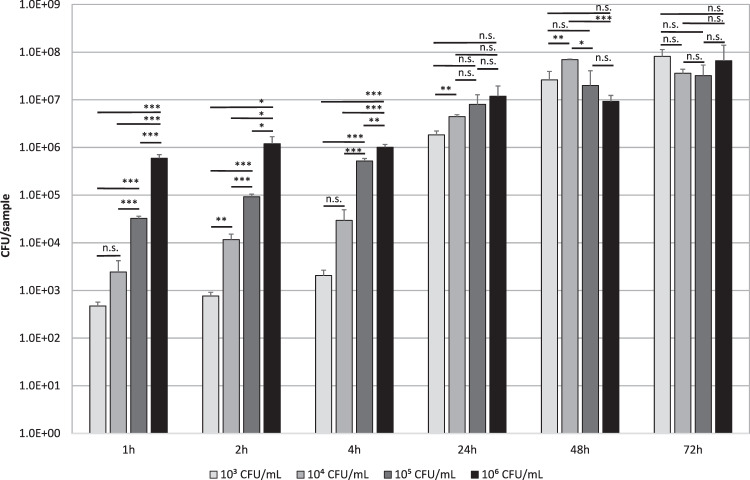
Influence of inoculum concentration on biofilm formation of *P. fluorescens* during 72 h incubation in LBM on borofloat coverslips. Inoculum concentrations of 10^3^, 10^4^, 10^5^, and 10^6^ CFU/mL were compared. Biofilm was quantified by colony forming units after biofilm separation and serial dilution. Data are given as mean ± standard deviation

The inoculum concentration plays an important role in the adherence of bacteria in the first 4 h of incubation. In this time, the inoculum concentration corresponded with the bacteria quantified on the substrate with a significantly different number of living bacteria isolated from biofilms with inoculum concentrations of 10^3^, 10^4^, 10^5^, and 10^6^ CFU/mL (Fig. 4). After 24 h, influence of the initial microbial concentration equalized and no considerable differences in bacteria count were detectable at 24, 48, and 72 h using different inoculum concentrations.

### Fluid management

**Fig. 5:**
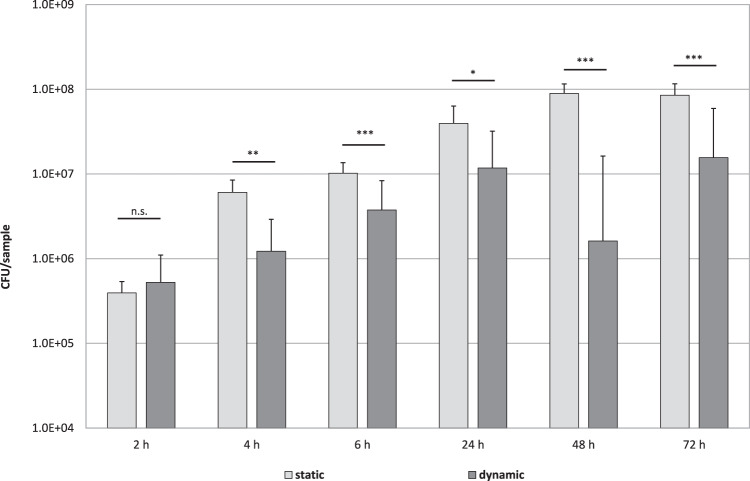
Influence of static and dynamic conditions on formation of *P. fluorescens* biofilm during 72 h incubation in LBM in 96-well MTP. Dynamic conditions were created with a wobble shaker at 200 rpm. Medium was changed after 24 and 48 h. Biofilm was quantified by colony forming units after biofilm separation and serial dilution as total CFU/well. Data are given as mean ± standard deviation

Using a wobble shaker to simulate dynamic growth conditions and to increase the shear stress, a reduced amount of adhering bacteria of *P. fluorescens* was observed compared to static conditions (Fig. 5). This effect was most evident after 48 h incubation.

**Fig. 6:**
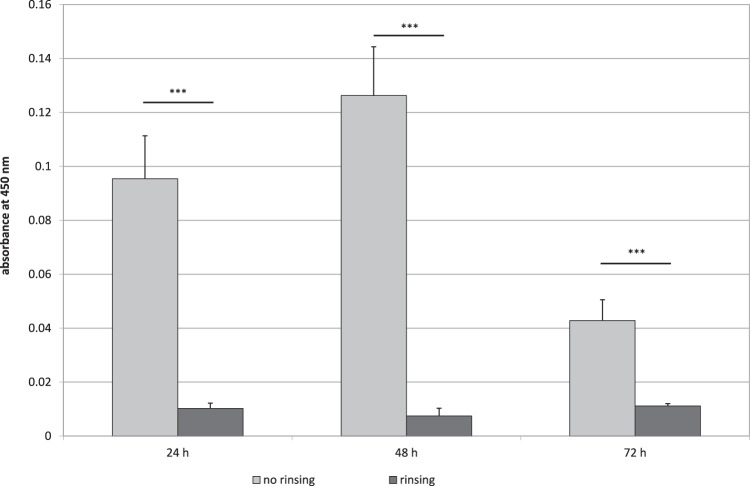
Influence of a rinsing step on formation of *P. fluorescens* biofilm during 72 h incubation in LBM, quantified by WST-1. The biofilm was rinsed once with LBM before quantification and during medium change after 24 and 48 h. Data are given as mean ± standard deviation

In another experiment for mimicking shear stress, the samples were rinsed with LBM before quantification and before medium change after 24 and 48 h. As demonstrated in Fig. 6, a significant reduction of biomass was detected when introducing a rinsing step during static incubation.

### Material properties

**Fig. 7:**
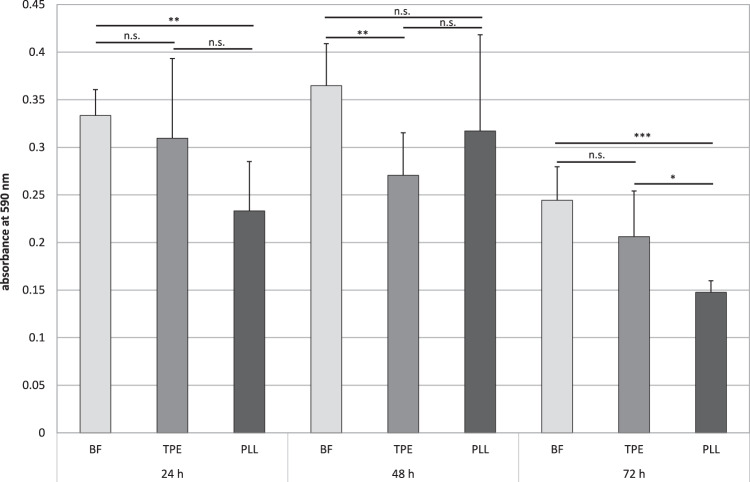
Influence of the substrate on formation of *P. fluorescens* biofilm during 72 h incubation on borofloat glass (BF), Nunc^TM^ Thermanox^TM^ polyester (TPE), and poly-L-lysin coated coverslips (PLL) in 1:100 LBM. Biofilm mass was quantified by crystal violet staining and subsequent absorbance measurement at 590 nm. Data are given as mean ± standard deviation

Incubation of *P. fluorescens* on borofloat glass, Nunc^TM^ Thermanox^TM^ polyester, and poly-L-lysin coated coverslips in 1:100 LBM pointed out the influence of the material on biomass formation (Fig. 7). The highest biomass at each time point was detected using borofloat glass with a maximum after 48 h incubation. Poly-L-lysin coated coverslips showed a reduced formation of biofilm compared to the other materials at 24 and 72 h of incubation, the maximum biomass was quantified at 48 h. Incubation of Nunc^TM^ Thermanox^TM^ polyester with *P. fluorescens* resulted in a decreasing biomass during the incubation period.

### Sample treatment for visualization

**Fig. 8:**
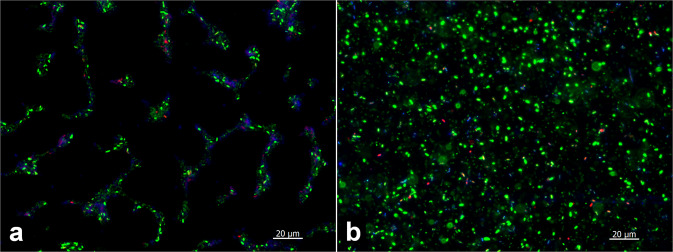
Influence of sample treatment on visualization of a 24 h *P. fluorescens* biofilm incubated on borosilicate glass coverslips in LBM 1:100 diluted in 0.9% NaCl. Whereas drying of the substrate during incubation and staining procedures with SYTO9/PI/CFW was partially allowed (**a**), it was consequently avoided in (**b**) during staining

Sample handling turned out as a critical step for visualization of in-vitro biofilm. Initial formation of a biofilm is vulnerable to drying processes, which could lead to misinterpretation of the adhering process. Removal of liquid during staining and washing of the whole MTP enabled individual wells to fall dry for a short time. Hence, microbial islands were observed when neglecting moisture management during sample preparation (Fig. 8a). This effect could be avoided by treating each well separately. In this way, samples were kept consequently with a liquid film, which resulted in an even distribution of microbes on the surface (Fig. 8b).

## Discussion

Cultivation of biofilms on material surfaces is influenced by a variety of experimental conditions. The design of biofilm experiments for material research has to be optimized with regard to the particular research task and critical parameters in this process have to be considered. In this study, the influence of different factors on biofilm formation was examined i.e. experimental time frame, nutrient supply, inoculum concentration, static and dynamic conditions, material properties, and sample treatment.

In aqueous medium, formation of a biofilm starts almost immediately by conditioning of the substrate with a layer of organic and inorganic particles [12]. This layer alters surface charge, potential and tension of the substrate and provides favorable conditions for bacterial anchorage [13]. After reversible adhesion of planktonic microbial cells to the conditioned surface, a number of cells remain immobilized and become irreversibly adsorbed. These sessile cells start dividing and a rapid exponential growth phase occurs, often resulting in mushroom-like structures. The production of EPM increases bonding between the cells and imparts mechanical stability of the biofilm. The composition of the matrix is species- and surface-dependent. It is a complex of secreted polysaccharides, proteins, nucleic acids from lysed cells, and absorbed nutrients and ions from the surrounding [2]. In this environment, microbes are effectively protected from harmful external conditions like antibiotics or desiccation.

In our study, adhesion of *P. fluorescens* on borosilicate glass substrate is already observed after 15 min incubation. Due to rinsing processes during sample preparation and staining, only firmly bound microbes were observed microscopically. The initial transport of microorganisms towards the substratum surface followed by adhesion is very fast. Different mechanisms play an important role in this process, like Brownian motion, gravitation, and diffusion [14]. Crouzet et al. observed a similar timeline in initial biofilm formation by *P. aeruginosa* with attached microbes after 20 min incubation [15]. After 2 h incubation, an evenly distributed layer of bound microorganisms was detected in our study. This experimental period of initial adhesion has to be considered for research focusing on e.g. material development studies to prevent initial adhesion of microorganisms to surfaces. Metabolic activity of the *P. fluorescens* biofilm in our experiments increased over a period of 16 h, reaching a plateau of accumulation after 16–24 h. These values are comparable with model systems described in the literature, where the plateau of accumulation in biofilms is achieved after 24 [16], 48 [17], or 72 h [18]. In the stationary phase of a mature biofilm, the rate of cell division equals the rate of cell death [13]. Enzymes like alginate lyase in the case of *P. fluorescens* are produced by the accumulated biofilm for polysaccharide breakdown and release of surface bacteria, which can colonize additional surfaces [13]. The timeline to reach this plateau of accumulation and the final composition of the extracellular matrix is dependent on species, nutrient availability and fluidic conditions [19]. When working with mature biofilms to investigate e.g. biofilm eradication strategies, it is important to determine the timeline when the plateau of accumulation is reached under the respective conditions to create a reproducible, controllable experimental biofilm.

The influence of nutrient supply on biofilm formation with regard to timeline and quality is crucial in material research. In our study, incubation in undiluted medium with high nutrient load resulted in a strong bacterial growth with biofilm floats and excessive formation of mucus. High nutrient availability in the medium leads to high cell growth rates followed by excessive mucus production and breakdown of the biofilm by enzymatic and mechanical processes [13]. Our results are in accordance with biofilm formation studies with *Acinetobacter baumannii*, where higher nutrient concentrations decreased biofilm accumulation and poor nutrient media increased biofilm production [20]. Studies on the influence of nutrient supply on biofilm formation in a microchannel system by Liu et al. demonstrated a strong nutritional effect on biofilm morphology [21]. With high nutrient load, the formation of thick, but loose structures was observed, that are highly sensitive to shear stress. In our study, best outcomes with formation of a stable biofilm on the material surface and without excessive mucus production were observed by using 1:10 diluted LB medium. Further reduction of nutrients resulted in slow bacterial growth and little formation of EPM. Kroukamp et al. [22] also demonstrated that a higher nutrient concentration leads to more biomass accumulation. However, this larger biomass is prone to more sloughing, which increases the variability of the biofilm biomass after the first sloughing events. The nature and amount of nutrients present may influence the morphology of bacteria, their surface, and adherence. In oligotrophic, aquatic environments with nutrient stress, organisms respond with enhanced adherence [19]. Therefore, a species dependent selection of medium and medium concentration is important for producing experimental biofilms.

Inoculum cell density plays an important role with regard to biofilm adherence studies. In our experiments, the influence of increasing inoculum concentrations on quantification of *P. fluorescens* in the biofilm was detectable only in the adherence phase of 4 h. After 24 h incubation, no considerable difference was observed in biomass formation with different initial numbers of microbes. In literature, inoculum concentrations in biofilm models vary to a large degree. For *Staphylococcus epidermidis* biofilm models in 96-well plates, a range from 10^3^ to 10^9^ CFU/mL was reported [23]. Cotter et al. observed a concentration-dependent consumption of dissolved oxygen in the medium in a static 96-well-plate model, resulting in a limited growth at high cell concentrations and alignment of biofilm formation after 4–6 h, independent from starting inoculum concentration [23]. Cell density-sensing molecules were described for *A. baumannii* and other bacterial pathogens [24, 25] for communication between bacterial cells during biofilm formation. These signaling molecules are part of the intercellular communication, known as quorum sensing, that regulates a number of processes during biofilm formation and maturation, like initial formation, control of population size, virulence, defending against other competitive microorganisms, avoiding toxic materials, and detachment [2, 12, 26]. When a plateau of accumulation in the biofilm is reached, in our studies after 16–24 h incubation, the number of microbes is balanced, independently from starting inoculum concentration.

Fluid management and rinsing procedures have strong influence on adherence, amount and shape of biofilm formation, and detachment processes [3, 27]. This could be confirmed in our studies, where rinsing and shaking reduced biomass formation. Fluid management in experimental biofilms should be adjusted to the research task, the material in question and to the natural occurrence of the biofilm investigated. A variety of biofilm models exist that differ in fluid management. Biofilm formation in MTP is the most popular model [10]. In this model, biofilm is grown in static conditions in the plate or on substrates placed into the plate. The main advantages of this user-friendly, straightforward model are low costs because of small volumes and little technical efforts. This system is ideal for screening purposes regarding antibiofilm substances, substrate modifications, or varying multiple parameters like growth media or atmosphere [9]. Because this model is batch reactor-like, the environment in the wells will change during the experiment, as nutrients are depleted and signaling molecules accumulate, unless the fluid is regularly replaced. Shear stress can only be applied to the system by shaking and rinsing. Many real-life biofilms are exposed to flow conditions and have constant supply of fresh nutrients [28]. So, other investigations may require specific hydrodynamic conditions, substratum composition, or large quantities of biomass. A number of flow displacement models exist with varying shear stress and nutrient distribution, like the modified Robins device, the Centers for Disease Control biofilm reactor, commercially available flow cells, drip flow reactors, or the rotating disc reactor [9]. In these “open” systems nutrients are continuously added and waste-products are removed.

To increase shear stress in MTP models, plates can be agitated. In our studies, increasing shear stress by shaking resulted in decreasing biomass compared to static cultures. The same effect was shown by Jiang et al. where increasing agitation at high speed enhanced dispersal of biofilms [29]. Similar results were also obtained for *Salmonella* spp. biofilms incubated in 96-well plates, where biofilm formation was significantly reduced by shaking [30]. In contrast, Donné et al. reported an increase in biofilm biomass with slow horizontal shaking at 25 rpm compared to incubation without shaking of *Escherichia coli* after 72 h [2]. They postulate a better biofilm growth at slow shaking speed due to a better nutrient and oxygen dispersion. Therefore, shaking velocity has to be carefully adjusted when agitating MTP models to obtain optimal biofilm growth.

Another influential factor on initial attachment and growth of biofilms are of course the material surface properties. Especially with regard to the major health complications following indwelling medical device infections, investigation into smart materials and coatings that can reduce bacterial attachment is the focus of modern antibiofilm strategies [31]. A number of material properties, like chemical composition, surface charge, roughness, hydrophobicity, or stiffness, influence the biofilm behavior [2, 31]. It was observed, that bacterial attachment accelerates with increasing roughness [12]. By tuning the hydrophobicity of a surface, bacterial adhesion can be either promoted or inhibited, because the preference of surface hydrophobicity differs among the bacterial species [31]. In our studies, differences of biofilm formation were seen between borofloat glass, Nunc^TM^ Thermanox^TM^ polyester, and poly-L-lysin coated coverslips with the highest biomass grown on borofloat glass. All tested materials are hydrophilic. Borofloat glass and Nunc^TM^ Thermanox^TM^ polyester are negatively charged in contrast to positively charged poly-L-lysin coated coverslips. The surface charge could be the reason for the observed differences in microbial adhesion.

Visualization of microbes adhering to surfaces is a very informative tool for studying biofilm formation processes. To reflect reality during this process we found that sample handling is very important. Especially in the initial state of biofilm formation, the organisms are very vulnerable to drying processes and observed “islands” could lead to misinterpretation of the adhering behavior. To overcome this problem, samples must be kept sufficiently moist during the entire sample preparation and dyeing processes. To our knowledge, this effect caused by sample handling during staining processes of initially adhering biofilms is not yet described in literature.

## Conclusion

Experimental biofilms are very important tools for understanding the underlying mechanisms of biofilm formation on material surfaces under controlled conditions. For designing intelligent medical biomaterials, these model systems can be successfully used to promote the effective fight against biofilms. In this study, a number of critical factors for cultivation of experimental biofilms in material research are pointed out to help in designing optimal experimental biofilm setups.

## Data Availability

All outcome data are available as representative images in the main text. The raw datasets generated and analysed during the current study are available from the corresponding author on reasonable request.
